# Belgian health-related data in three international databases

**DOI:** 10.1186/0778-7367-69-6

**Published:** 2011-11-01

**Authors:** K Vanthomme, D Walckiers, H Van Oyen

**Affiliations:** 1 OD Public Health and Surveillance, Scientific Institute of Public Health, J. Wytsmanstraat 14, 1050 Brussels, Belgium

**Keywords:** Belgium, databases, health status indicators, public health

## Abstract

**Aims of the study:**

This study wants to examine the availability of Belgian healthcare data in the three main international health databases: the World Health Organization European Health for All Database (WHO-HFA), the Organisation for Economic Co-operation and Development Health Data 2009 and EUROSTAT.

**Methods:**

For the indicators present in the three databases, the availability of Belgian data and the source of these data were checked.

**Main findings:**

The most important problem concerning the availability of Belgian health-related data in the three major international databases is the lack of recent data. Recent data are available for 27% of the indicators of the WHO-HFA database, 73% of the OECD Health Data, and for half of the Eurostat indicators. Especially recent data about health status (including mortality-based indicators) are lacking.

**Discussion:**

Only the availability of the health-related data is studied in this article. The quality of the Belgian data is however also important to examine.

The main problem concerning the availability of health data is the timeliness. One of the causes of this lack of (especially mortality) data is the reform of the Belgian State. Nowadays mortality data are provided by the communities. This results in a delay in the delivery of national mortality data. However several efforts are made to catch up.

## Introduction

The aim of this report is to examine the availability of Belgian data in three existing international health databases: the World Health Organization (WHO) European Health for All Database (HFA-DB) [1], the Organisation for Economic Co-operation and Development (OECD) Health Data 2009 [2], and EUROSTAT [3].

## Methods

Three questions were addressed:

1) Are Belgian data available for each indicator?

2) Are the available data up-to-date?

3) Where should the data come from (national or international source)?

For each indicator in the three databases, the availability was checked for all the years included in the dataset. If data were available for recent years (since 2006), the indicator was considered as 'available' and 'recent'. If data were available, but only up to and including 2005 or earlier, the indicator was considered as 'outdated'. If there were no Belgian data at all for an indicator, the indicator was considered 'not available'. The 'not available' and 'outdated' indicators taken together are named 'missing indicators'. The examination of the availability in the WHO and OECD databases was done in December 2009, which means that for the WHO Health for All Database the version of August 2009 was used, and for the OECD Health Data the version of November 2009. Updates afterwards were not taken into account for this report. The availability in the Eurostat database was examined in January 2010. Not the whole dataset was taken, only demographic data and data about health, which are both part of the domain 'Population and social conditions' within the Eurostat database.

For our third question, the source of the Belgian data was examined. Three possible sources were distinguished: data coming from national sources, data originating from international organisations or studies, and data from a Belgian source, but compiled and calculated by an international organisation or study. In the latter case, if Belgian data were missing or outdated, a small comparison was done with four neighbouring countries. The availability of the indicator in Belgium was compared with the availability in France, Germany, The Netherlands, and the United Kingdom. If data were available for the majority (three or four) of these countries, the source was defined as national and the problem of unavailability or timeliness was said to occur in Belgium. If the neighbouring countries did not have data either, it was considered that the problem lay in the international organisation.

The focus of this report thus lies on the availability of Belgian health data in three core international databases. Nothing is said about the quality of these data, although this is a very important subject which needs to be examined in a next step.

## Results

### World Health Organization, European Health for All Database

The Health for All Database is a database of comparable and up-to-date basic health statistics. It has started in the mid-1980s, and contains information from 1970 onwards. The dataset is updated twice a year, with a request for data to national sources in September. This dataset contains 600 indicators for 53 countries. It contains demographic information, data on the health status (mortality, morbidity, maternal and child health), information about non-medical determinants of health (lifestyle and environment), and healthcare information (resources and utilisation). The data come from country experts, WHO/Europe's technical programmes and partner organisations such as OECD.

For less than one third (26.5%) of the 600 indicators, recent data are available in the database for Belgium (Table 1). The problem is not the availability itself but the timeliness of the data. For 70% of the indicators there are no Belgian data for 2006 or later. The most important problem concerns the mortality-based indicators. This domain accounts for 42% (252/600) of the indicators and has no recent available data. It represents 57% (252/441) of the missing indicators in the WHO Database. For most of these mortality indicators, the latest available year is 2004. The domain lifestyle does not have a good score either, with almost 90% missing data, but the impact of this domain is not important (only 5% of the missings indicators) because of the small number of indicators it contains (4% of the 600 indicators). Only the indicators concerning morbidity, disability and hospital discharges on the one hand, and environment on the other hand, have an availability of more than 50%. The domain of morbidity, disability and hospital discharges has however the second largest share of missings, namely 12% of the total missings, due to the big number of indicators it contains (19% of the 600 indicators).

**Table 1:** Availability of Belgian data in the World Health Organization, European Health for All Database

		*N variables (%)*					
		
Domain	Total N	Available and recent data		Outdated data		No data available	
Demographic and socio-economic indicators	36	16	*(44%)*	20	*(56%)*	0	*(0%)*
Mortality-based indicators	252	0	*(0%)*	249	*(99%)*	3	*(1%)*
Morbidity, disability and hospital discharges	116	64	*(55%)*	45	*(39%)*	7	*(6%)*
Lifestyle	26	3	*(11.5%)*	20	*(77%*	3	*(11.5%)*
Environment	23	12	*(52%)*	11	*(48%)*	0	*(0%)*
Healthcare resources	64	30	*(47%)*	28	*(44%)*	6	*(9%)*
Healthcare utilisation and expenditure	52	22	*(42%)*	29	*(56%)*	1	*(2%)*
Maternal and child health	31	12	*(39%)*	17	*(55%)*	2	*(6%)*

Total	600	159	*(26.5%)*	419	*(70%)*	22	*(3.5%)*

Table 2 shows how many indicators of the domains have (or should have) a national source, and how many an international source. It also gives an idea of the (un-) availability of data coming from national and international sources.

**Table 2:** Availability of Belgian data in the World Health Organization, European Health for All Database, by type of source

		*N variables*			
		
Domain		Total	Available and recent data	Outdated data	No data available
Demographic and socio-	National	27	14	13	0
economic indicators	International	9	2	7	0
Mortality-based indicators	National	241	0	238	3
	International	11	0	11	0
Morbidity, disability and	National	106	54	45	7
hospital discharges	International	10	10	0	0
Life styles	National	19	3	13	3
	International	7	0	7	0
Environment	National	12	10	2	0
	International	11	2	9	0
Health care resources	National	64	30	28	6
	International	0	0	0	0
Health care utilization and	National	40	18	21	1
expenditure	International	12	4	8	0
Maternal and child health	National	29	12	16	1
	International	2	0	1	1

Total	National	538	141	376	21
	International	62	18	43	1

Table 2 also includes the 38 'mixed sources', which means data coming from a national source but compiled and/or calculated by an international organisation. For 15 of the 33 indicators with a mixed source and a problem of availability of (recent) data, it was concluded that there was a problem at national level, and for 18 at international level. All five indicators with recent available data and a mixed source were placed under the heading 'international source'.

In total, 90% (538/600) of the indicators in the WHO Health for All Database (should) have a national source. Only for a fourth of these indicators data are available for Belgium, while for 4% there are no data at all. The major part, 70% of the indicators, has data which are outdated. The same tendency is seen in the international data. 71% of those indicators has no recent data for Belgium.

The pattern of table one continues, the major problem lies with mortality-based indicators. For this dimension, almost all data (241/252: 96%) come from a Belgian source, and there are no recent data available at all. This dimension accounts for 61% (241/397) of the missing national data, and 25% (11/44) of the missing international data. The second dimension with a large share of missing data is morbidity, disability and hospital discharges, which accounts for 13% (52/397) of the missing national data. This is related to the big number of indicators it contains (20% of the 538 indicators)

### Organisation for Economic Co-operation and Development (OECD) Health Data 2009

The OECD Health Data is a comprehensive database including indicators on the healthcare systems of the OECD member countries. It offers the most comprehensive source of comparable statistics on health and health systems across OECD countries. It is an electronic database, for which the request for data to national sources takes place in February, and which is released annually in June. OECD Health Data 2009 is produced in collaboration with IRDES [4]. The dataset contains information about health status (mortality and morbidity), healthcare information (resources and utilisation), economic data (expenditure, financing, economic references, social protection), data about the pharmaceutical market (sales and consumption), non-medical determinants of health (lifestyle and environment), and demographic data.

The availability of the OECD Health Data is much better than the availability of data in the Health for All Database. For 73% of the 3942 indicators recent Belgian data are available (Table 3). The main problem of the missing data is the timeliness. 16% of the data is not up-to-date. This is especially the case for indicators concerning expenditure on health (42% of the missings), and health status (34% of the lacking data), which contains mortality which was also problematic in the Health for All Database.

**Table 3:** Availability of Belgian data in the OECD Health Data 2009

		% *(N variables)*					
		
Domain	Total N	Available		Outdated		Not available	
Health status	394	28	*(7%)*	364	*(92%)*	2	*(1%)*
Healthcare resources	106	79	*(75%)*	14	*(13%)*	13	*(12%)*
Healthcare utilisation	571	563	*(99%)*	6	*(1%)*	2	*(0%)*
Long-term care resources and utilization	63	13	*(20.5%)*	3	*(5%)*	47	*(74.5%)*
Expenditure on health	2141	1687	*(79%)*	108	*(16%)*	346	*(5%)*
Healthcare financing	139	86	*(62%)*	35	*(25%)*	18	*(13%)*
Social protection	96	17	*(18%)*	77	*(80%)*	2	*(2%)*
Pharmaceutical market	249	228	*(91.5%)*	9	*(3.5%)*	12	*(5%)*
Non-medical determinants of health	29	3	*(10%)*	26	*(90%)*	0	*(0%)*
Demographic references	57	56	*(98%)*	1	*(2%)*	0	*(0%)*
Economic references	97	97	*(100%)*	0	*(0%)*	0	*(0%)*

Total	3942	2857	*(73%)*	643	*(16%)*	442	*(11%)*

As in the WHO Database, the largest part of indicators has a national source (3543/3942: 90%, table 4). There were 106 indicators with a 'mixed source', which have all been placed under a national source after comparison with the four other countries.

**Table 4:** Availability of Belgian data in the OECD Health Data 2009, by type of source

		*N variables*			
		
Domain		Total	Available	Outdated	Not available
Health status	National	344	5	337	2
	International	50	23	27	0
Healthcare resources	National	103	76	14	13
	International	3	3	0	0
Healthcare utilisation	National	571	563	6	2
	International	0	0	0	0
Long-term care resources and utilisation	National	63	13	3	47
	International	0	0	0	0
Expenditure on health	National	2099	1687	81	331
	International	42	0	27	15
Health care financing	National	139	86	35	18
	International	0	0	0	0
Social protection	National	0	0	0	0
	International	96	17	77	2
Pharmaceutical market	National	207	198	0	9
	International	42	30	9	3
Non-medical determinants of health	National	17	3	14	0
	International	12	0	12	0
Demographic references	National	0	0	0	0
	International	57	56	1	0
Economic references	National	0	0	0	0
	International	97	97	0	0

Total	National	3543	2631	490	422
	International	399	226	153	20

A first look at table 4 learns that the indicators from a Belgian source are in terms of percentage less lacking than indicators with an international source. The indicators concerning the expenditure on health account for the greatest deal of missing indicators (454/1085: 42%). The second problematic dimension is as in the WHO dataset, health status (including mortality as in WHO).

Looking at the missing indicators with a national source, the same pattern is seen: the most problematic dimension is expenditure on health (412/912: 45% of the missing national indicators), before health status (339/912: 37%). Two important remarks should be made. First, 249 of the 412 missing expenditure data coming from a national source are not compulsory (total long-term care expenditure and expenditure by age and gender). Second, 39 of the expenditure variables are not relevant for the Belgian situation (expenditure on services not allocated by function) and for 35 variables the requested information is not available for Belgium (private investment in health services, health services of other industries (private current) and price index) because the Belgian methodology and the international one differ on this point. Excluding these variables, the percentage of missing expenditure data originating from a national source decreases to 5% (95/1776).

Furthermore, for 12 of the 139 national healthcare financing indicators, the requested information is not available in Belgium because there is no such financing in Belgium (health expenditure by financing agents/schemes: rest of the world), which reduces the percentage of missing variables to 32% instead of 38%.

The large amount of outdated non-medical determinants can be explained by the fact that the source of the data is the Health Interview Survey, which is conducted every 3-5 years (last data sent to the international organisations refer to the 2004 survey).

Concerning the indicators having an international source, especially social protection is problematic, as it accounts for 46% (79/173) of the missing international indicators.

### Eurostat

Eurostat is the statistical office of the European Union [5]. It provides statistics at European level to the European Union which makes it possible to compare countries and regions. It offers data about several themes such as economy, industry and transport. Only a small part of the dataset is included in this report. The indicators about demography and health are included, both being part of the domain 'population and social conditions'. Indicators about demography and health which are only collected at European (e.g. the number of accidents at work by type of injury and severity) or regional level (e.g. age-specific death rate), but not on national level, are also excluded. The request for these data from national sources takes place every August.

In total there are 158 indicators dealing with demography and health (Table 5). For only 7% there are no data available for Belgium and for half of the indicators recent data are available. For 42% of the indicators there are data for Belgium, but they are outdated.

**Table 5:** Availability of Belgian data in the Eurostat database

		% *(N variables)*					
		
Domain	Total N	Available and recent data		Outdated data		No data available	
Demography	35	22	*(63%)*	12	*(34%)*	1	*(3%)*
Public Health	110	52	*(47%)*	55	*(50%)*	3	*(3%)*
Health and safety at work	13	6	*(46%)*	0	*(0%)*	7	*(54%)*

Total	158	80	*(51%)*	67	*(42%)*	11	*(7%)*

The dimension demography has a better availability of data than the two dimensions about health. In the dimension public health which contains indicators about causes of death, expenditure, resources and patients, the main problem is the timeliness of data. In the dimension about health and safety at work, for half of the indicators, there are no data available for Belgium at all. One must however consider the much lower number of indicators in this dimension.

When looking at the sources for Belgium, we can conclude that the great majority (89%) of the data come from national sources (Table 6). There were six indicators with a 'mixed source' which are placed under international source after comparing with the four aforementioned countries.

**Table 6:** Availability of Belgian data in the Eurostat database, by type of source

		*N variables*			
		
Domain		Total	Available and recent data	Outdated data	No data available
Demography	National	35	22	12	1
	International	0	0	0	0
Public Health	National	98	40	55	3
	International	12	12	0	0
Health and safety at work	National	7	6	0	1
	International	6	0	0	6

Total	National	140	68	67	5
	International	18	12	0	6

Data originating from an international organisation or study are more frequently available for Belgium than data coming from a national source (12/18: 67%, against 68/140: 48.5%). On the other hand, data coming from a national source have a much lower percentage of data not available (5/140: 3.5%) than data from an international source (6/18: 33%). We should however take into account the low number of indicators with an international source.

### International comparison

Missing data, and lack of recent data, are apparently an important problem for Belgium. Some data both from national and international sources/studies (which then apply to data for several countries) are missing or out-of-date, as well as data coming from national sources but calculated by international organisations.

To have an idea of how good or bad the score of Belgium is at the international level, a small comparison was made. The availability of data for the WHO indicators was also checked for France, Germany, The Netherlands and the United Kingdom. All the indicators of mortality were excluded for this comparison because we already know that Belgium is not up-to-date with the delivery of these data. Although stable conclusions cannot be made upon this rough comparison, it still gives an idea of the situation.

Table 7 shows the percentage of available data for the different domains in the WHO database for Belgium, and a mean score for the four neighbouring countries. Belgium scores worse than the other countries on demographic and socio-economic indicators, healthcare resources, healthcare utilisation and expenditure, and maternal and child health. For these last dimensions however, Belgium is not the worst student of the class. Belgium has the highest score of the five countries for availability of data about morbidity, disability and hospital discharges, and scores are also higher than average for data about environment and lifestyle. The availability of data about lifestyle is however not good at all, only 11.5% of the indicators can be documented with data.

**Table 7:** International comparison of availability of data in the WHO database*: Belgium versus France, Germany, The Netherlands and the United Kingdom

	Percentage of available data	
	
Domain	Belgium	Mean international score (range)
Demographic and socio-economic indicators	44	75 (69-78)
Morbidity, disability and hospital discharges	55	42.5 (34-48)
Lifestyle	11.5	4 (0-19)
Environment	52	45 (26-74)
Healthcare resources	47	51 (0-84)
Healthcare utilisation and expenditure	42	59.5 (40-71)
Maternal and child health	39	44 (26-65)

Total	48	44 (24.5-58)

* indicators of mortality excluded

The total availability of data for Belgium in the WHO dataset without mortality-based indicators seems to be good. The total score is higher than the average score of the four neighbouring countries. The range of these countries is however large. When excluding the outlier (value 24.5), the mean score for the three remaining countries is an availability of 51% (44-58) which is higher than the availability for Belgium. In this case the availability of Belgium is equal to the lower range.

## Discussion and conclusions

First of all, one should notice that this report only describes the availability of the data. Besides timeliness, other aspects of the quality of data (e.g. accuracy, comparability, usefulness and relevance) need to be explored in a next step.

Regarding the availability of data for Belgium, there is a great difference between the WHO Health for All Database, the OECD Health Data, and Eurostat. While data for Belgium are only available for 27% of the indicators in the WHO Health for All database, this amounts to 51% in the Eurostat database, and to 72% in the OECD Health Data database (Table 8) (even 78% after correction for the irrelevant and non compulsory indicators). An explanation for this difference, particularly between the WHO and OECD databases, is the number of indicators related to health status, and especially the proportion of mortality-based indicators in the whole dataset. For WHO, 42% of the database consists of mortality-based indicators, while only 10% of the indicators of OECD are related to health status (containing mortality and morbidity).

**Table 8:** Overview of availability problems for Belgian data in OECD, WHO and Eurostat databases, by type of source

	OECD (N = 3942)	WHO (N = 600)	Eurostat (N = 158)
*No data available*	11% (N = 442)	3.5% (N = 22)	7% (N = 11)
Data from national counterparts	95%	95%	45.5%
Data from international sources/studies	5%	5%	55.5%
*Data available but timeliness problem*	16% (N = 643)	70% (N = 419)	42% (N = 67)
Data from national counterparts	76%	90%	100%
Data from international sources/studies	24%	10%	0%
***Total: no recent data available***	27% (N = 1085)	73.5% (N = 442)	49% (N = 78)
Data from national counterparts	84%	90%	92%
Data from international sources/studies	16%	10%	8%

When looking at the sources, in all three datasets about 90% of the data comes from a national source.

One conclusion that can be made based upon this exploration of the availability in three international databases is the lack of recent Belgian mortality, or broader, health status data. All three datasets no recent data are available about mortality. This dimension accounts for a great proportion of the missing indicators, especially in the WHO database. Indicators concerning health status account for 69% (304/441) of the missing indicators in the WHO HFA-DB, and (after correction) for 46% (366/797) of the missing indicators in the OECD Health Data. The reform of the Belgian State has had consequences on the provision of health information and data. Over the last decades, Belgium has become a federal state. In the 1980s, collecting mortality data became a competency of the communities and since then the medical information on the forms of death certificates is encoded at community level [6]. This results in a delay in provision of national mortality data but during the last years many efforts have been done to shorten the delay. The recent European regulation in this domain should enhance the capacity to have data on causes of mortality with a delay of less than 2 years [7].

In the OECD Health Data database there is another dimension which scores badly, namely expenditure on health (21% of the missings). A comparison with WHO and Eurostat databases of expenditure on health is not very appropriate because of the low number of indicators about expenditure in these databases (OECD: 2141 (or 1853 after correction), WHO: 21, and Eurostat: 13).

Belgium should consequently urgently make an effort to catch up, especially on data about mortality. The availability of Belgian non-mortality data in the WHO dataset was quite good in comparison with neighbouring countries. If Belgium could solve this problem, a great deal of the lacking health data could be made available.

The OECD, EUROSTAT and WHO-Europe have been collecting data on monetary and non-monetary health care resources for many years and they agreed on a new joint data collection which has been launched in 2010. The most important goal is to reduce the burden of data collection for the national authorities responsible for the provision of statistical information to the international organisations. Moreover, a joint effort will increase the use of international standards and definitions and improve the consistency of data reported by international organisations. This new joint data collection would initially cover key variables related to: 1) human resources in health; and 2) physical/technical resources. In order to achieve consistency in data submissions to the three international organisations, one Focal Point for the joint data collection on non-monetary healthcare statistics had to be designated in 2009 [8]. In Belgium, this Focal Point will be assisted by a coordination group with representatives of the federal and federated entities [9].

Initiatives have already been taken in the past to coordinate all activities concerning health statistics and to provide up-to-date information. To reflect reforms in the structures of the Belgian state, the composition of the Superior Council of Statistics was amended by the Royal Decree of March 31, 1998 (Belgian Official Gazette May 6, 1998) [10]. This Council is a coordinating council, intra- and interfederal by its composition: representatives of federal, community and regional authorities, parastatal and subordinate institutions. Besides this, in March 2000, a protocol for data exchange between federal authorities and authorities responsible for health policy was signed in order to coordinate the collection of information [11]. The crucial need for a national focal point with representatives from different institutions was also mentioned in a report published in 2008 in the framework of the implementation process of the ECHI indicators (European Community Health Indicators) [12]. The aim of the ECHIM (European Community Health Indicators and Monitoring) Joint Action (2009-2011) is to advance health monitoring throughout Europe by developing relevant and comparable health indicators and by making them available in the EU and its Member States, as well as in other European countries.

Although lots of health data are available in Belgium, they are until now not integrated into a national health information system. Health data from different regions and communities have to be aggregated in order to get national data. An overview of the most important healthcare databases has been made by the KCE in 2006 [13]. A problem is the comparability of data from different sources: different definitions, reference periods and calculations are used. This also makes it difficult to compare data over time. There is thus an urgent need for coordination between all concerned authorities to improve the situation. Special attention should be paid to the development of data-exchange systems, so that timely information can be provided.

The Belgian Focal Point for the data collection on non-monetary healthcare statistics should first make an inventory of possible data sources, with information on their timeliness, periodicity and representativeness. Inventoried sources could be clustered into five groups: disease-specific registers, annual statistical reports, administrative databases, surveys and studies. The definition, sources and methods (sampling, calculation,...) underlying the data should be documented. Problems/weaknesses related to data collection should be listed. Different problems could for instance arise when a source is used for another purpose than the original one (codes of diagnoses which are reimbursed at higher level could be "overused"). Another very important problem related to routine data collection systems is lack of quality assessment for some data sources.

The Focal Point should afterwards choose the most appropriate source and try to fill in the gaps. An information system should be developed to centralise the information. The establishment of a regularly maintained national database with aggregated data at national and regional levels, available online, would increase the availability, accessibility and use of health information by different kinds of users (policy makers, healthcare professionals, researchers, students,...). This database should also contain metadata to allow users to correctly interpret data, and include data presentation tools. Hopefully this valorisation of data will lead to an increase in their quality and also in the data exchange between all involved partners.

## Competing interests

The authors declare that they have no competing interests.

## Authors' contributions

KV participated in the design of the study, performed the analysis and drafted the manuscript. DW and HVO made substantial contributions to the study design, data interpretation and analysis and helped to draft and revise the manuscript. All authors read and approved the final manuscript.
